# The Popeye Domain Containing Genes and Their Function in Striated Muscle

**DOI:** 10.3390/jcdd3020022

**Published:** 2016-06-15

**Authors:** Roland F. R. Schindler, Chiara Scotton, Vanessa French, Alessandra Ferlini, Thomas Brand

**Affiliations:** 1 Developmental Dynamics, Harefield Heart Science Centre, National Heart and Lung Institute, Imperial College London, Hill End Road, Harefield UB9 6JH, UK; r.schindler@imperial.ac.uk (R.F.R.S.); v.french@imperial.ac.uk (V.F.); 2 Department of Medical Sciences, Medical Genetics Unit, University of Ferrara, Ferrara 44121, Italy; sctchr@unife.it (C.S.); fla@unife.it (A.F.)

**Keywords:** atrioventricular block, cyclic AMP, cardiac arrhythmia, membrane trafficking, membrane protein, limb-girdle muscular dystrophy, sinus bradycardia

## Abstract

The Popeye domain containing (POPDC) genes encode a novel class of cAMP effector proteins, which are abundantly expressed in heart and skeletal muscle. Here, we will review their role in striated muscle as deduced from work in cell and animal models and the recent analysis of patients carrying a missense mutation in *POPDC1*. Evidence suggests that POPDC proteins control membrane trafficking of interacting proteins. Furthermore, we will discuss the current catalogue of established protein-protein interactions. In recent years, the number of POPDC-interacting proteins has been rising and currently includes ion channels (TREK-1), sarcolemma-associated proteins serving functions in mechanical stability (dystrophin), compartmentalization (caveolin 3), scaffolding (ZO-1), trafficking (NDRG4, VAMP2/3) and repair (dysferlin) or acting as a guanine nucleotide exchange factor for Rho-family GTPases (GEFT). Recent evidence suggests that POPDC proteins might also control the cellular level of the nuclear proto-oncoprotein c-Myc. These data suggest that this family of cAMP-binding proteins probably serves multiple roles in striated muscle.

## 1. Introduction

The Popeye domain containing genes encode a class of 3′-5′-cyclic adenosine monophosphate (cAMP) effector proteins, which is abundantly expressed in cardiac and skeletal muscle [1]. The second messenger cAMP is an ancient signaling molecule, which is present in all living organisms from bacteria to humans [2,3]. It is a universal indicator of environmental stress and causes a large number of different physiological reactions. An intricate network of cAMP-producing enzymes (adenylate cyclases), effector proteins, cAMP-degrading enzymes (phosphodiesterases), as well as scaffolding proteins (e.g., AKAPs) are present in eukaryotic cells [4]. This protein network ensures that the production of cAMP is compartmentalized, allowing the activation of cAMP effector proteins and their downstream targets in a spatio-temporally-controlled manner [5].

In eukaryotes, three well-characterized classes of cAMP effector proteins are known: protein kinase A (PKA), exchange factor directly activated by cAMP (EPAC) and the cyclic nucleotide-gated ion channels (CNGC) [6]. Recently, the cyclic nucleotide receptor involved in sperm function (CRIS) has been identified as the fourth cAMP effector protein and plays a role in sperm maturation and motility [7]. The Popeye domain containing (POPDC) genes encode a fifth class of cAMP-effector proteins, which displays a prominent expression in striated muscle tissue [8]. Work in model organisms revealed important roles of POPDC genes for cardiac pacemaking and conduction and for skeletal muscle regeneration and maintenance of structure and function [9,10,11]. Recently, the first patients carrying mutations in POPDC genes were reported, who suffer from muscular dystrophy and cardiac arrhythmia [12]. In this review article, we will introduce the POPDC gene family, describe their subcellular localization and discuss their functional roles in skeletal muscle and heart. We will update the reader on striated muscle proteins that are interacting with POPDC proteins. Finally, we will discuss some recent evidence suggesting an important role of POPDC proteins in the control of cell proliferation and wound healing.

## 2. Structural Elements of the Popeye Domain Containing Proteins

Parallel attempts by two groups to isolate novel genes with a cardiac-restricted expression pattern led to the discovery of the first member of the POPDC family, which was named *POPDC1* and blood vessel epicardial substance (*BVES*) [1,13]. Subsequently, two cDNAs were identified in vertebrates that displayed significant sequence homology to *POPDC1* and, therefore, were named *POPDC2* and *POPDC3* [1]. However, POPDC genes display limited homology to other genes.

**Figure 1:**
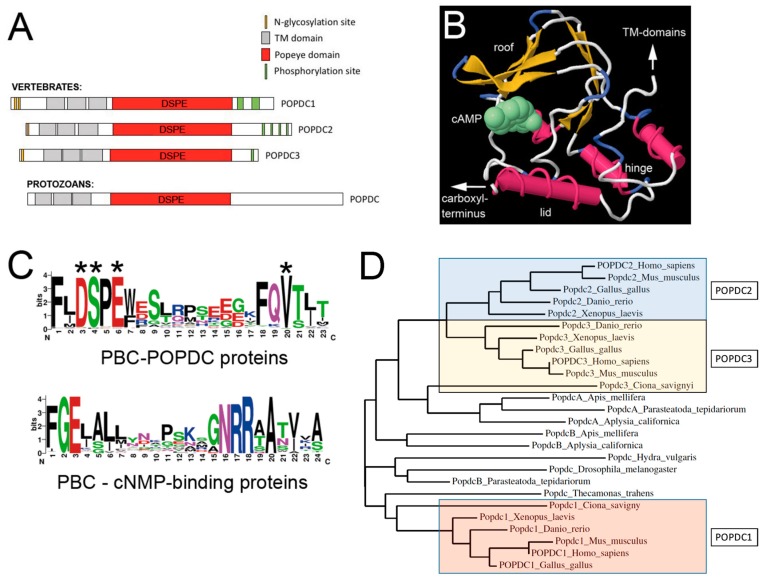
The Popeye domain containing protein family. (**A**) Graphic depiction of the domain structure of POPDC proteins. POPDC proteins consist of a short extracellular amino terminus, which is subject to *N*-glycosylation, followed by three transmembrane domains and the Popeye domain. The approximate location of the invariant DSPE motif, which is part of the phosphate-binding cassette (PBC), is indicated. The carboxy terminal part of the proteins is variable in size, differs between family members and contains a cluster of serine/threonine residues, which are subject to phosphorylation in response to β-adrenergic stimulation [16]. The structure of a protozoan POPDC protein has an overall identical structure, suggesting an ancient origin of the POPDC protein family. (**B**) Structural model of the Popeye domain of POPDC1. The 3D model depicted here was generated using the First Glance in Jmol website [18]. The structural elements (roof, hinge and lid) of the Popeye domain have been identified based on their similar location in other cAMP-binding domains [6]. (**C**) Comparison of the PBC of POPDC proteins and that of canonical cyclic nucleotide monophosphate (cNMP)-binding proteins. In POPDC proteins, cAMP-binding involves the ultra-conserved sequence motifs DSPE and FQVT. Experimental evidence for the involvement in cAMP-binding has been obtained for D^200^, S^201^, E^203^ and V^217^ (asterisks). The canonical cAMP-binding cassette strongly diverges from the one present in POPDC proteins. Sequence data utilized for the generation of the logo for the Popeye domain were retrieved from the PFAM entry of the Popeye domain [19]. For the protein sequences of non-POPDC cAMP binding proteins, the data were downloaded from Prosite (PS00889; CNMP_BINDING_2) [20,21]. (**D**) A phylogenetic tree of vertebrate and invertebrate POPDC protein sequences. The vertebrate Popdc proteins cluster into three groups: POPDC1, POPDC2 and POPDC3. The POPDC1 and POPDC3 proteins of the urochordate *Ciona savignyi* appear to be orthologous to their respective vertebrate counterparts, supporting a model of two gene duplication events during chordate and vertebrate evolution, respectively. Popdc proteins in other invertebrates do show similarity to either Popdc1 or Popdc2/3, and thus, protein ancestry cannot be easily deduced from this analysis. The tree was generated with the help of the Phylogeny online platform [22].

POPDC genes encode membrane proteins, which consist of about 290–370 amino acids and have predicted molecular weights of 34–42 kDa. In contrast, the native proteins isolated from striated muscle tissue display significant larger molecular weights (55–70 kDa). Interestingly, the electrophoretic mobility of POPDC1 differs, when isolated from cardiac or skeletal muscle. In cardiac muscle, the major immunoreactive POPDC1 protein bands run at a molecular weight of 55–58 kDa, while in skeletal muscle, POPDC1 has an apparent molecular weight of 70 kDa [8,14]. It is thought that this may be based on tissue-specific differences in *N*-glycosylation [1,15], but could also be due to other types of posttranslational modifications or be the result of tissue-specific alternative splicing. The extracellularly-localized amino terminus of a POPDC protein is short (20–40 residues) and followed by three transmembrane domains (Figure 1A). The intracellular part of the protein consists of the evolutionarily-conserved Popeye domain, which acts as a cAMP-binding domain. Distal to the Popeye domain is the carboxy terminus region, which is variable in size and differs between family members, but displays interspecies conservation [1]. The carboxy terminus of POPDC proteins contains clusters of serine/threonine residues, which get phosphorylated in response to β-adrenergic stimulation [16].

Structural predictions of the Popeye domain suggest it may largely resemble the secondary structure of the cAMP-binding domain of PKA [10]. However, the two proteins otherwise lack sequence homology. A three-dimensional model of the Popeye domain was generated on the basis of similarity to the cAMP-binding domain of the cyclic AMP receptor protein (CRP) from *Streptomyces coelicolor*, which displays 20% sequence identity over 149 residues [10]. Using this structural model (Figure 1B), the majority of the conserved residues were mapped to the phosphate-binding cassette (PBC) or to immediately surrounding parts of the Popeye domain [10]. The PBC of POPDC proteins do not resemble the consensus sequence of other cAMP-binding proteins (Figure 1C). Importantly, divergent PBCs are also present in a number of other proteins, where the small molecule that binds to this domain is not cAMP, but for example, heme or chlorophenolacetic acid [17]. 

We therefore need to address whether POPDC proteins do indeed bind cAMP. There is plenty of evidence in support of a cAMP-binding property of POPDC proteins: (1) The recombinant Popeye domain of mouse POPDC1 binds cAMP with high affinity, which is in the range of PKA [10]. In the same assay, the binding affinity for cGMP is approximately 45-fold lower than for cAMP (cAMP IC_50_: 118.4 ± 7.1 nM; cGMP IC_50_: 5.27 ± 0.68 µM), which suggests that POPDC proteins have a high specificity for cAMP and therefore being able to discriminate between closely-related cyclic nucleotides. (2) Assays in *Xenopus* oocytes and HEK293 cells, which are based on the interaction of POPDC1 and the ion channel TREK-1 (see below), demonstrate that protein-protein interaction is modulated by cAMP [10]. (3) Mutagenesis of several evolutionary conserved residues, which are located in the PBC of POPDC1 (D200, E203 and V217) and POPDC2 (D184), cause a strong reduction in the affinity for cAMP [10]. (4) There is also the identification of patients that carry a mutation in the PBC of POPDC1 (S201F, see below) [12] causing a 50% reduction in cAMP-affinity. This mutation is associated with striated muscle disease, providing further evidence for the regulation of POPDC proteins by cAMP. Moreover, this finding also clearly demonstrates the biological significance of cAMP-binding of POPDC proteins.

We can therefore conclude that cAMP signaling in eukaryotes is mediated by at least two different groups of effector proteins, those with a canonical PBC (PKA, EPAC, CNGC and CRIS) and POPDC proteins, which harbour a divergent PBC.

## 3. Evolution of the Popeye Domain Containing Proteins

While in vertebrates, three POPDC genes are found, only two genes are present in lower chordates (tunicates, lancelets) and echinoderms. POPDC genes are also present in invertebrates. In molluscs and insects, most species have two POPDC genes. Interestingly, *Drosophila* has only a single POPDC gene [23], whereas the POPDC gene is absent in *Caenorhabditis elegans*. In vertebrates, *POPDC1* and *POPDC3* are tandem-localized, and in humans, both genes are present on chromosome 6q21, while *POPDC2*, is located at 3q13.33. A similar genomic organization with two genomic loci is also found in other vertebrate species.

POPDC proteins have evolved early during animal evolution. Recent GenBank analysis identified POPDC proteins also in protozoans (NCBI: XP_013760779.1) (Figure 1A). Surprisingly, despite several billions of years of evolutionary time that separate the vertebrates and protozoans, the overall structure appears to be very similar, suggesting strong evolutionary pressure to maintain the sequence and the resulting protein structure. No evidence was obtained so far for the presence of POPDC proteins in plants or fungi.

A phylogenetic tree has been constructed using the protein sequences of human POPDC1–3 and of the vertebrate model organism (mouse, chick, *Xenopus* and zebrafish) (Figure 1D). In addition, representatives of major clades, basic chordates (*Ciona*), insects (*Drosophila*, *Apis*), chelicerates (*Parasteatoda*), molluscs (*Aplysia*), coelenterates (*Hydra*) and protozoa (*Thecamonas*), were aligned, and sequence divergence representing phylogenetic distance was visualized using a phylogenetic tree. Vertebrate POPDC family members cluster into three groups representing Popdc1, Popdc2 and Popdc3. Significantly, Popdc1 is well separated from Popdc2 and Popdc3, which are more similar to each other. The presence of three genes is probably due to two gene duplication events, as previously proposed [24]. The first gene duplication event generated *Popdc1* and *Popdc3* and seems to predate the common ancestor of chordates, as it is shared by all vertebrates, urochordates and cephalochordates [25]. The second duplication event probably took place during vertebrate evolution and generated *Popdc2*, which is unique to vertebrates and localized on a different chromosome than *Popdc1* and *Popdc3* [1]. Popdc genes in non-chordate invertebrates appear to have evolved separately from the chordate genes, and therefore, orthology cannot be determined.

As mentioned above, there is no significant sequence homology between POPDC proteins and any of the other eukaryotic cAMP effector proteins. However, some of the bacterial cAMP-regulated transcription factors, catabolite activator protein (CAP) and cAMP receptor protein (CRP), display weak, but significant sequence homology, which formed the basis for the development of a structural model of the Popeye domain (Figure 1B) [12].

## 4. The POPDC Gene Family Encodes Membrane Proteins Predominantly Expressed in Skeletal Muscle and Heart

Expression of POPDC genes in the mouse embryo has been reported for *Popdc1* and *Popdc2* [1,11,26,27]. Expression of *Popdc2* was mostly monitored with the help of a LacZ knock-in (KI) reporter allele [27]. Expression was first detectable between Embryonic Day (E) 6.0 and 7.5 in the extraembryonic mesoderm and in the heart. At E8.5, *Popdc2* was found in the tubular heart, pericardium and amnion. At E10.5, expression was present in the mesothelial layer of the coelomic wall, extraembryonic tissues, the heart and muscle precursors in the head and body wall. At E12.5, expression was present in the coelomic wall, heart and skeletal muscle tissues. At this stage, expression in the heart was confined to the myocardium and was absent from epi- and endocardium. Smooth muscle cells of the developing gastrointestinal tract and bladder were also stained. Expression of *Popdc1* was studied by enzyme histochemistry in mouse embryos carrying a LacZ KI allele and by whole mount in situ hybridisation [11]. With the help of both techniques, expression in the myocardium, skeletal muscle and smooth muscle of gut and bladder was described. As for *Popdc2,* expression of the *Popdc1* LacZ allele was confined to the myocardium, and expression was absent in epi- and endocardium and their derivatives. In contrast, Smith *et al*. reported POPDC1 protein expression in coronary artery smooth muscle cells [26]. Differences in detection limits, antibody specificities and tissue-specific epitope expression may explain the divergent outcomes of these expression studies.

**Figure 2:**
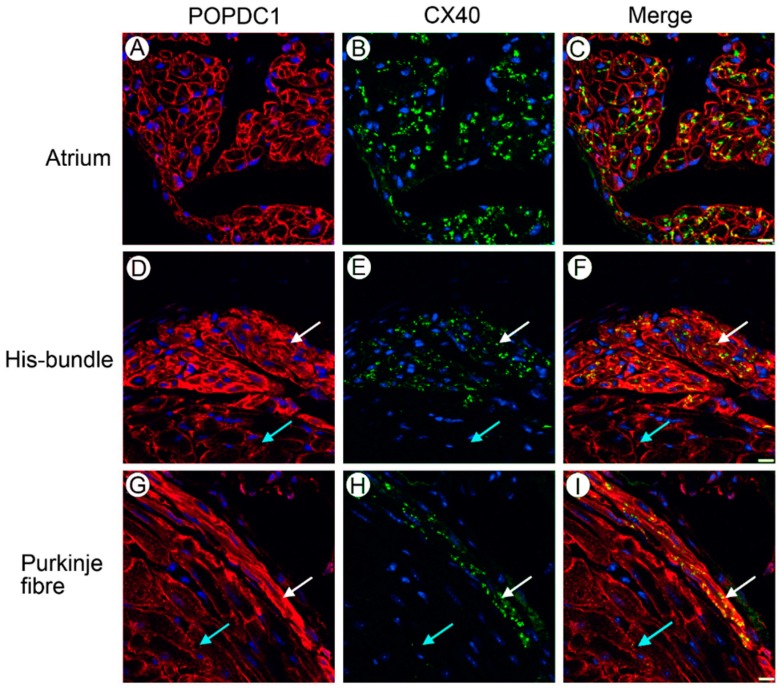
Differential expression of POPDC1 in the murine heart. POPDC1 is expressed at higher levels in the (**A**–**C**) atria, (**D**–**F**) His bundle and (**G**–**I**) Purkinje fibres in comparison to the ventricular working myocardium. Frozen sections of mouse myocardial tissue were immunohistochemically stained with antibodies directed against POPDC1 (red signal) and, as a marker of ventricular conduction tissue, Cx40 (green signal). The white arrows in (**D**–**I**) label ventricular conduction tissue, while the turquoise arrows label ventricular working myocardium.

In the adult, POPDC proteins are expressed strongly in cardiac and skeletal muscle. In the heart, the expression level of POPDC1 is higher in the atria than in ventricles, while POPDC2 is expressed equally in both chamber types [10,27,28]. High levels of POPDC1 and POPDC2 expression are seen in the sinoatrial (SAN) and atrioventricular (AVN) nodes [10,27]. Moreover, the ventricular conduction tissue displays higher levels than the working myocardium (Figure 2). In addition to striated muscle tissue, POPDC proteins are also strongly expressed in smooth muscle tissue lining the digestive tract, the uterus and the bladder [10,27,28]. Moreover, sensory neurons in the dorsal root ganglia, as well as neurons in the central nervous system express POPDC1 and POPDC3. Expression has also been described in epithelial cells of the digestive tract and the cornea [27,29].

In cardiac myocytes, POPDC1 and POPDC2 are localized to the plasma membrane and are present at the intercalated disk, lateral membrane and in the t-tubuli system [10,27,28]. In addition, POPDC proteins have also been localized to the nuclear envelope [30,31]. It will be interesting to define their function in the nuclear compartment and their possible roles in diseases that are associated with the nuclear envelope, such as dilated cardiomyopathy (DCM), Emery-Dreifuss muscular dystrophy (EDMD) and limb-girdle muscular dystrophy (LGMD) [28].

A peculiar observation is the finding that POPDC proteins are also present in the nucleoplasm. POPDC proteins accumulate in the nucleus, when a cDNA is transfected that encodes a protein lacking the transmembrane domains. Interestingly, endogenous POPDC1 protein is nuclear-localized in activated satellite cells, but the protein is cytoplasmic after myoblasts have fused and myotubes have formed [32]. The functions of nuclear POPDC proteins have not been elucidated yet, but an involvement in muscle regeneration has been proposed [11,32].

## 5. Muscle Regeneration Is Retarded in *Popdc1* Mouse Mutants

A *Popdc1* null mutant allele was engineered by replacing the coding region of the first exon with a nuclear LacZ reporter gene [11]. The mutant animals were subjected to isoproterenol-induced cardiac hypertrophy and cardiotoxin-induced skeletal muscle wounding. While the hypertrophic response in the myocardium was similar between mutant and wildtype, skeletal muscle regeneration appeared to be retarded in the null mutant mice, as indicated by the prolonged expression of myogenic transcription factors and the histology of the regenerating muscle. Importantly, *Popdc1* expression is induced in activated satellite cells [11]. At present, however, the precise role of POPDC1 during muscle regeneration has not been fully understood. Furthermore, a reduction in the ability to cope with myocardial ischemia-reperfusion injury was reported for *Popdc1* null mutant mice [33], suggesting a broader role of *Popdc1* in response to striated muscle injury.

## 6. Mouse *Popdc1* and *Popdc2* Mutants Develop a Stress-Induced Sinus Node Bradycardia

A mouse *Popdc2* null mutant allele was engineered in a similar way as for *Popdc1* by placing a LacZ reporter gene into the first coding exon [10]. Expression analysis of the LacZ reporter gene of both mutant alleles, as well as immunohistochemical detection of the endogenous proteins revealed a prominent expression in the cardiac conduction tissue, including the SAN and AVN (Figure 2) [10]. This observation prompted an investigation of cardiac pacemaking and conduction by implanting a telemetric ECG devise into null mutants. Cardiac conduction was normal in resting mouse mutants. However, a stress-induced sinus node bradycardia developed after subjecting null mutants to physical or emotional stress or after isoproterenol injection [10]. This phenotype was observed not only in *Popdc2*, but also in *Popdc1* null mutants. Significantly, the pathological phenotype developed in both mutants in an age-dependent manner. While the pathological phenotype was not present in young animals, null mutant mice at 5–8 months of age developed a severe stress-induced bradycardia with episodes of sinus node dysfunction [10].

## 7. *Popdc2* Depletion in Zebrafish Is Associated with Atrioventricular Block and Muscular Dystrophy

Like other vertebrates, three POPDC genes are present in zebrafish. Similar to mammalian and avian hearts, expression of zebrafish *popdc1* and *popdc2* is first detectable in the heart just shortly before cardiac contraction starts [9,12]. Expression of *popdc1* and *popdc2* in embryonic skeletal muscle is observed once myotubes are beginning to differentiate in the myotome. Expression of *popdc2* in tail musculature is transient and is absent by 3 dpf; however, it remains present in the heart and in facial muscles [9]. In the adult, expression of both *popdc1* and *popdc2* is detected in heart and skeletal muscle [9,12]. Depletion of *popdc2* with morpholino oligonucleotides targeted to the splice acceptor and donor sites caused a strong loss-of-function phenotype. In the heart, a massive pericardial effusion developed, which is a sign for embryonic heart failure. When the morpholino concentration was reduced, the pericardial effusion became smaller, while a second cardiac phenotype emerged, i.e., an atrioventricular (AV)-block. Initially, morphant hearts were beating regularly until 4–5 days post fertilization (dpf), when a 2:1 or 3:1 AV-block phenotype was observed [9]. As the morphants grew older, hearts were observed with extensive sinus pauses or with an electrically silent ventricle. In skeletal muscle, a severe muscular dystrophy phenotype was present [9]. It is thought that the malformation of the myotendinous junctions (MTJ), which probably does not allow proper anchoring of the myofibrils into the MTJ, contributes to the dystrophy phenotype. In addition to the aberrant tail musculature in the *popdc2*-depleted embryos, a dysmorphic facial musculature is observed. A similar striated muscle pathology including AV-block and muscular dystrophy was also observed in the case of *popdc1* morphants [12].

## 8. A POPDC1^S201F^ Mutation Causes Limb-Girdle Muscular Dystrophy and Cardiac Arrhythmia

**Figure 3:**
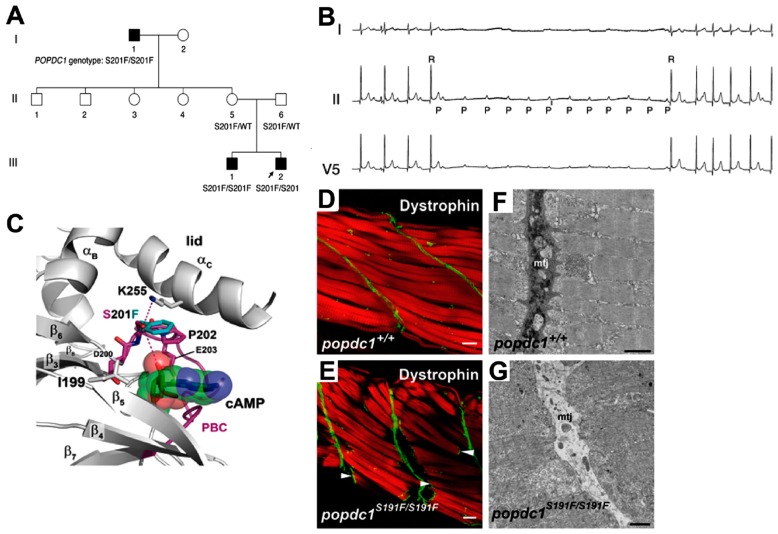
The POPDC1^S201F^ missense mutation causes cardiac arrhythmia and muscular dystrophy. (**A**) Pedigree of an Italian family homozygous for the POPDC1^S201F^ mutation. The index patient (III-2, arrow) and his brother (III-1) suffer from AV block, while the grandfather (I-1) has both, AV block and LGMD. (**B**) Holter electrocardiogram of the index patient displaying an episode of a paroxysmal AV block. (**C**) 3D model of the Popeye domain. Only the phosphate-binding cassette (PBC) is shown. Two hydrogen bonds are formed by S201, but these are predicted to be lost when the S201 residue is replaced by a phenylalanine (turquoise) affecting the ligand-binding affinity of the Popeye domain. (**D**–**G**) Sections through the zebrafish tail musculature. (**D**,**E**) Confocal analysis of actin (phalloidin staining, red channel) and dystrophin (green channel) in trunk skeletal muscle of (**D**) WT (*popdc1^+/+^*) and (**E**) homozygous zebrafish mutant (*popdc1^S191F/S191F^*) at 5 dpf. Homozygous mutants develop lesions in skeletal muscle. Muscle fibres detach from the MTJ and retract. Arrowheads indicate the presence of dystrophin immunoreactivity at the end of the ruptured fibres, suggesting that the fibres have lost connection to the MTJ, but probably remain physically intact. (**F**,**G**) TEM analysis of trunk skeletal muscle of (**F**) WT and (**G**) homozygous *popdc1^S191F/S191F^^F^* mutant zebrafish embryos at 5 dpf. The MTJ in the mutant is characterized by a lack of electron-dense material. Panels (**A**–**G**) are reproduced from [12] with permission.

As discussed in the previous sections, null mutations and depletion of *Popdc1* and *Popdc2* in animal models caused severe pathologies in the heart and skeletal muscle. However, until recently, it was uncertain whether POPDC gene mutations were disease-associated in humans and whether patients carrying mutations develop pathologies similar to those observed in model organisms. Recently, a family carrying a recessive missense (S201F) mutation in *POPDC1* and suffering from limb-girdle muscular dystrophy (LGMD) was identified (Figure 3A). The patients display an early onset AV-block coupled with a late onset mild muscular dystrophy (Figure 3B) [12]. This novel LGMD was recently classified as LGMD2X [34].

LGMD is a heterogeneous group of muscular dystrophies, which primarily affect hip and shoulder muscles [35]. There are both autosomal dominant (LGMD1) and autosomal recessive (LGMD2) subtypes known. To date, 31 different genetic loci of LGMD have been identified, most of them inherited in an autosomal recessive fashion. LGMD-causing genes encode proteins that belong to several different cellular pathways, including sarcolemmal glycoproteins (dystroglycan and sarcoglycans), scaffolding proteins (caveolin-3 (CAV3)), membrane repair and vesicle trafficking proteins (dysferlin (DYSF)) and nuclear envelope proteins (lamin A/C (LMNA)) [35].

A significant number of the LGMD gene mutations not only cause a muscular dystrophy, but also cardiac phenotypes, including a progressive reduction in ventricular ejection fraction, heart failure and cardiac arrhythmia [36]. Interestingly, in many cases, the pathology in the heart is early onset and more severe than in skeletal muscle [37].

The mutated residue S201 in POPDC1 is ultra-conserved and part of the DSPE motif of the PBC (Figure 1C and Figure 3C). S201 is thought to be directly involved in nucleotide binding. Indeed, measuring cAMP-binding of the mutant protein revealed a significant reduction [12]. In order to generate an animal model for this human missense mutation, a homologous mutation was introduced into the orthologue in zebrafish. The resulting homozygous *popdc1^S191F/S191F^* mutant fish also displayed muscular dystrophy (Figure 3D–G) and cardiac arrhythmia and, therefore, largely resembled the mutant phenotype observed in patients [12]. The human S201F mutation is rare, and screening of an additional 104 patients with cardiac arrhythmia and/or muscular dystrophy did not reveal any additional carrier [12].

Recently, *POPDC1* was identified as a hub gene for atrial fibrillation (AF) using a weighted co-expression network analysis [38]. However, no *POPDC1* mutations have yet been identified in AF patients. *POPDC1* has been recently identified as a novel genetic determinant of the QT interval and QRS duration [39]. Expression of POPDC genes is downregulated in heart failure (HF) [40]. The reduction in POPDC gene expression and the resulting loss of POPDC protein expression in the failing hearts may contribute to the myocardial arrhythmia, which is prevalent in heart failure. However, more work in animal models is required to define whether the reduction in POPDC expression is causally related to the disease phenotype in heart failure.

A number of different functions and associated pathologies in striated muscle tissue have been associated with POPDC genes both in animal models and in patients (Table 1). However, there seems to be also species-specific differences. Both, in zebrafish morphants and mutants, an AV-block has been described. Similarly, patients with a S201F missense mutation of POPDC1 display an AV-block. In contrast, *Popdc1* and *Popdc2* null mutants have a stress-induced sinus bradycardia, while an AV-block has not yet been described. These phenotypic differences in model organisms and patients may be related to the vastly different heart rates in mice compared to zebrafish and humans [41].

## 9. Interaction Partners of POPDC Proteins

### 9.1. Zonula Occludens-1

The first interaction partner of POPDC proteins that was identified is the PDZ protein Zonula occludens-1 (ZO1) [42]. ZO-1 functions as a scaffolding protein of tight junctions and contains several protein domains, with which it interacts with other proteins, such as the tight junction proteins claudin and occludin, actin and several signaling proteins [43]. The downregulation of POPDC1 in cultured epithelial cells reduces transepithelial resistance and junctional protein localization at the plasma membrane, which suggests that POPDC1 contributes to the establishment and/or maintenance of epithelial integrity [42]. In cardiac myocytes, ZO1 binds the carboxy terminus of the gap junction protein Connexin43 (Cx43) and thereby controls gap junction plaque size [44]. Thus, the POPDC1/ZO-1 interaction could be of significance with respect to cardiac conduction. While POPDC proteins are expressed throughout the sarcolemma, some antibodies directed against POPDC1 or POPDC2, specifically, recognize an expression domain in the intercalated disk region [26,45,46]. Interestingly, cAMP signaling via PKA and EPAC modulates membrane trafficking of Cx43 and stimulates gap junction neo-formation in neonatal cardiac myocytes [47]. Modulation of PKA and EPAC has also been implicated in myocardial protection and prevention of post-ischemic cardiac arrhythmia, which is also observed in *Popdc1* null mutant mice [33,48].

### 9.2. VAMP2/3, GEFT and NDRG4

With the help of a yeast-two-hybrid assay, three interacting proteins, namely vesicle-associated membrane protein 3 (VAMP3) [49], N-myc downstream regulated gene 4 (NRDG4) [50] and guanine nucleotide exchange factor T (GEFT) [51], were identified. VAMP3 and the closely-related protein VAMP2 are SNARE proteins, which act as components of the vesicle docking and fusion complex [52]. VAMP proteins are involved in membrane trafficking of a number of different receptor proteins through the recycling endosome. Thus, VAMP2 and VAMP3 have both been implicated in GLUT4 translocation in cardiac and skeletal muscle [53,54]. Interestingly, VAMP2 has been found to be expressed in satellite cells and is upregulated after cardiotoxin injection [55]. Thus, the protein-protein interaction of POPDC1 with VAMP2 and VAMP3 could be of relevance in the context of the observed regeneration defect in the *Popdc1* null mutant [11].

Moreover, VAMP2/VAMP3 also play an important role in the control of cell adhesion and cell migration, mainly through the modulation of integrin receptor recycling [56,57]. This function of VAMP proteins could also be of relevance in striated muscle regeneration. Moreover, integrin receptor recycling may also be required for the maintenance of the MTJ in zebrafish embryos, which is strongly affected in *popdc1* mutants and *popdc2* morphants [9,12]. The stability of the MTJ depends on two membrane receptor complexes, i.e., the dystrophin glycoprotein complex (DGC) and the integrin receptor [58,59]. Both receptor complexes bind the basement membrane protein laminin 2 and, thereby, anchor the muscle cell to the MTJ [60]. Loss-of-function mutations in each of these proteins cause a phenocopy of the MTJ phenotype in POPDC1 mutants. Thus, it will be important to work out the epistatic relationship of the different proteins acting in this context. Interestingly, the role of cAMP in MTJ formation is currently unknown. It is noteworthy that cAMP levels in astrocytes modulate membrane trafficking of VAMP3. Therefore, POPDC proteins could be involved in recruiting VAMP3 to the plasma membrane [61]. A role for POPDC1 in membrane trafficking is also supported by the fact that Popdc1 interacts with NDRG4 [50]. Knockdown of both *Popdc1* and *Ndrg4* affects VAMP3 vesicle docking. In addition, loss of *Ndrg4* also affects cell migration via fibronectin/integrin receptor internalization [50].

GEFT, a guanine nucleotide exchange factor (GEF) for the Rho-family GTPases Rac1 and Cdc42, has also been identified as an interaction partner of POPDC1 [51]. GEFT is expressed in cardiac and skeletal muscle. Overexpression of GEFT causes a rearrangement of the microfilaments and affects the morphology and migratory activity of cells. Forced expression of a construct, encoding a POPDC1 mutant protein lacking the transmembrane domains, affected the activation of Rac1 and Cdc42 without affecting RhoA [51]. Furthermore, transfection of the same construct into C2C12 cells attenuated muscle differentiation. How POPDC1 modulates GEFT is currently unknown, and several mechanisms have been proposed, including direct control of GEFT activity, sequestration of GEFT and, therefore, preventing it from activating downstream effectors or recruiting it to a particular subcellular localization [51,62]. GEFT has also been implicated in skeletal muscle regeneration [63]. Moreover, it also needs to be mentioned that GEFT controls neurite outgrowth [64]. Interestingly, in *Popdc1* and *Popdc2* null mutants, SAN myocytes display structural abnormalities, which might be due to aberrant GEFT activity [10].

### 9.3. TREK-1

The presence of POPDC proteins in the plasma membrane of cardiac myocytes and the high expression levels in the cardiac conduction system prompted a search for ion channels that interact with POPDC proteins. This led to the identification of the potassium channel TWIK-related K^+^ channel 1 (TREK-1) as a novel POPDC interacting protein [10]. TREK-1 is a member of the two-pore domain potassium channel (K2P) family, which is modulated by membrane stretch, intracellular pH, temperature, phosphorylation and protein–protein interactions [65]. Co-expression of any one of the three POPDC proteins and TREK-1 in *Xenopus* oocytes resulted in an approximately two-fold higher current, when compared to oocytes in which TREK-1 was expressed alone [10]. The increase in TREK-1 current can be explained by an enhanced membrane trafficking of the ion channel in the presence of POPDC proteins [10]. This effect was modulated by cAMP-binding to POPDC proteins and was lost in the presence of increased cAMP levels.

It is thought that the carboxy terminus of POPDC1 is directly interacting with TREK-1, as demonstrated by a GST pull-down experiment using carboxy-terminal POPDC1 as bait [10]. Moreover, a bi-molecular FRET sensor consisting of POPDC1-CFP and YFP-TREK-1 demonstrated direct physical interaction [10]. Moreover, the FRET ratio obtained under baseline conditions decreased after raising the intracellular cAMP levels, suggesting that cyclic nucleotide-binding induces an allosteric effect in the Popeye domain, which affects the interaction with TREK-1.

Interestingly, TREK-1 in myocytes interacts with β_IV_-spectrin and ankyrin G [66], which are involved in membrane targeting and complex formation of a number of cardiac ion channels [67]. A cardiac-specific knockout of *Kcnk2* encoding TREK-1 displays a stress-induced sinus bradycardia similar to the one observed in *Popdc1* and *Popdc2* null mutants [68], which means that the sinus bradycardia phenotype of POPDC mutants could at least be partially explained by the loss of the POPDC-TREK-1 interaction.

In support of a role of POPDC proteins in regulating membrane trafficking is the recent finding that the mutant POPDC1^S201F^ protein in the muscle tissue of the LGMD2X patients displayed a reduction in membrane localization and increased perinuclear levels. POPDC2 was localized in a similar way in the mutant muscle. Surprisingly, the increase of cAMP levels caused a reduction in membrane localization of TREK-1 in the presence of POPDC1^S201F^, whereas a reduction in the cAMP-binding affinity of POPDC1^S201F^ caused a reduction in the membrane transport of itself and of POPDC2. These data suggest that the effect of cAMP on membrane trafficking of POPDC1 and associated proteins is complex. Therefore, more work is required to unravel how cAMP-binding of POPDC proteins affects protein-protein interaction and membrane trafficking.

### 9.4. Caveolin-3, Dystrophin and Dysferlin

The sarcolemmas of skeletal and cardiac muscle are complex structures and contain caveolae, which cover the entire cell surface, and t-tubules, which are plasma membrane invaginations into the centre of muscle cells. Both structures contribute to membrane compartmentalization. Different ion channels accumulate specifically in caveolae and t-tubules [69]. Interestingly, caveolae have been implicated in mechanosensing and mechanoprotection [70]. Both caveolae and t-tubules have been linked to caveolin-3 (CAV3), the major membrane protein of caveolae in striated muscle [71]. *Cav3* null mutant mice display muscular dystrophy and cardiomyopathy. They lack caveolae and display an abnormal t-tubular structure in skeletal muscle, whereas a reduction in caveolar number was observed in cardiac muscle [72,73].

Recently, an interaction of POPDC1 and CAV3 has been reported [33]. The binding site for CAV3 has been mapped to a motif located in the lid structure of the Popeye domain (Figure 1B). *Popdc1* null mutants display a reduction in the number and an increase in the diameter of caveolae [33]. Interestingly, amongst other muscle and heart conditions, mutations of CAV3 have been linked to an autosomal dominant form of LGMD (LGMD1C) [34]. In *Popdc1* null mutants, altered Ca^2+^ transients were observed [33]. Thus, the loss of POPDC proteins may result in abnormal caveolar and t-tubular organisation and functions, which may affect physiological parameters, such as ion currents. Changes in compartmentalization may be responsible for the abnormal stress-induced arrhythmia observed in *Popdc1* and *Popdc2* null mutants [10]. However, in muscle biopsies of LGMD2X patients carrying the POPDC1^S201F^ mutation, membrane localization of CAV3 was not affected [12]. While the demonstration of a physical interaction between POPDC1 and CAV3 and overlapping pathological phenotypes in patients and in model organisms is compelling, the biological significance of this protein-protein interaction is currently uncertain and requires more research.

Muscle has the ability to repair an injury by two principle mechanisms: (1) large and significant injuries that causes fibre degeneration and necrosis are repaired by satellite cell-mediated regeneration; (2) micro-injuries, on the other hand, are repaired via a mechanism that involves the protein dysferlin, which recruits other proteins, such as annexins, to the site of membrane damage, leading to a rapid sealing of the sarcolemma [74]. Skeletal muscle fibres of one of the patients carrying the homozygous POPDC1^S201F^ mutation display focal loss of the sarcolemma [12]. This phenotype has been previously observed in patients carrying mutations in anoctamin 5 (*ANO5*) [75], *CAV3* [76] and in *Dysf* null mutant mice [77]. Mutations in *DYSF* have been associated with LGMD type 2B and Miyoshi myopathy [78]. DYSF-mediated membrane repair is also required in cardiac muscle, with its deficiency leading to cardiomyopathy [79].

POPDC1 and dysferlin are interacting proteins. They are co-localized and co-immunoprecipitated after co-transfection [12]. However, in muscle biopsies of LGMD2X patients carrying the POPDC1^S201F^ mutation, membrane localization of dysferlin was not affected [12]. Thus, despite the physical interaction and presence of membrane discontinuities in muscle tissue of LGMD2X patients, the biological significance of this protein-protein interaction is currently not fully understood.

Dystrophin (DYS) is a major cytoskeletal protein localized at the subsarcolemmal sarcolemma in cardiac and skeletal muscle. It forms a link between the cytoskeleton and the sarcolemmal dystrophin glycoprotein complex (DGC). The DGC consists of α and β-dystroglycan, sarcoglycans, sarcospan, dystrobrevin, syntrophins and neuronal nitric oxide synthase (nNOS) [80]. Dystrophin mutations are associated with Duchenne (DMD) and Becker-type muscular dystrophy (BMD). The disease phenotype in the case of BMD is usually milder and more slowly progressing. DMD is characterized by progressive loss of functional striated muscle tissue, causing muscular dystrophy and dilated cardiomyopathy with rhythm defects [81]. Most patients die before they reach the age of 25 [82].

Dystrophin is an interaction partner of POPDC1. Both proteins are co-localized and co-immunoprecipitated after co-transfection [12]. However, in muscle biopsies of LGMD2X patients, which carry the POPDC1^S201F^ mutation, membrane localization of dystrophin was not altered [12]. Therefore, at present, the biological role of this interaction is unknown.

### 9.5. c-Myc

A yeast two-hybrid (Y2H) assay using a human placenta library identified PR61α as a novel POPDC1-interacting protein [83]. PR61α is a regulatory subunit of protein phosphatase 2A (PP2A), which is critical for c-Myc degradation [83]. POPDC1 forms a complex with PR61α and c-Myc. RNAi-mediated knockdown of *Popdc1* in cell lines caused an increase in c-Myc levels, whereas forced expression caused a decrease. This suggests that POPDC1 controls c-Myc levels in cells by promoting degradation through complex formation with c-Myc, PR61α and the PP2A catalytic subunit [83]. Interestingly, the PR61α interaction domain was mapped to the carboxy terminus of POPDC1, which is not conserved between POPDC family members, suggesting that this interaction is probably unique to POPDC1. Presently, we do not know whether complex formation of POPDC1 with c-Myc, PR61α and the PP2A catalytic subunit is also observed in striated muscle tissue. If this would be the case, the POPDC1-mediated modulation of c-Myc levels could have an important impact on myoblast proliferation and differentiation and, therefore, on muscle regeneration. C-Myc can block myogenic differentiation even in the presence of the forced expression of myogenic transcription factors, such as MyoD and myogenin [84]. Similarly, c-Myc has been implicated in perinatal cell proliferation of cardiac muscle [85,86]. It also plays a role in cardiac hypertrophy and heart failure induction [87,88]. It will be important to test whether deregulated c-Myc expression is also present in striated muscle tissue of *Popdc1* null mutant mice. Such data would be relevant to define the molecular basis for the regeneration phenotype in *Popdc1* null mutants [11].

In summary, in recent years, a number of different proteins have been shown to interact with POPDC proteins, most of which are localised to the plasma membrane as transmembrane proteins or as membrane-associated proteins. However, with c-Myc, a nuclear interaction partner has now been identified, which suggests that POPDC proteins act probably in different subcellular compartments (Table 2).

## 10. Outlook

Since the discovery of POPDC proteins in 1999/2000, significant progress in our understanding of their role has been made [1,13]. Until very recently, it was uncertain whether the cardiac arrhythmia and muscular dystrophy phenotypes, which are present in animal models, occur in patients. However, the identification of LGMD patients with a strong cardiac phenotype and who carry a point mutation in *POPDC1* clearly demonstrates the significance of POPDC mutations in muscle disease. They also show the significance of cAMP-binding of POPDC proteins for normal muscle function. However, the disease spectrum caused by mutations in POPDC genes needs to be better defined. Since mutations in POPDC genes are obviously rare, it will also be necessary to define the role of POPDC genes in acquired heart muscle disease, such as heart failure, atrial fibrillation and ischemia-reperfusion.

**Figure 4:**
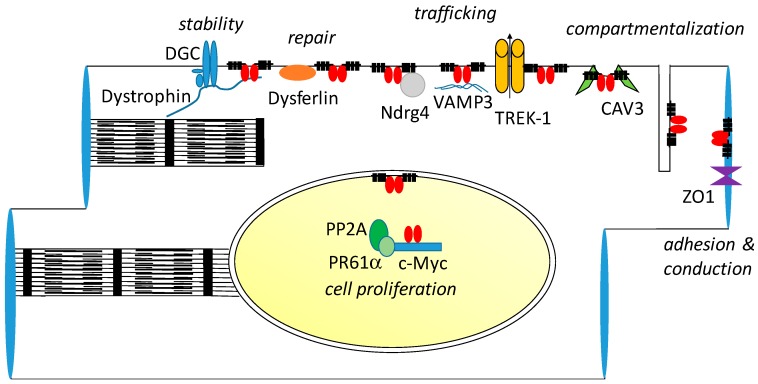
POPDC proteins participate in multiple protein complexes and subcellular compartments. Shown here is a schematic depiction of the different protein complexes of which POPDC1 is a part. Most of the interacting proteins are localized to the sarcolemma. However, Popdc1 and Popdc2 are also localized to specialized membrane compartments, such as caveolae, t-tubuli, nuclear envelope, nucleoplasm and intercalated disk. While a ventricular cardiac myocyte has been depicted here, some of the protein complexes have not been characterized in this cell type. The interacting proteins in the nuclear envelope and t-tubules have not yet been identified.

Surprisingly, in cardiac and skeletal muscle cells, POPDC proteins are not only present at the sarcolemma (Figure 4), but also in caveolae, t-tubules, intercalated disk, nuclear envelope and the nucleoplasm. It is likely that in each subcellular compartment, a specific set of proteins are forming complexes with POPDC proteins, and defining these complexes will be an important task in the coming years, resulting in a better understanding of the role of POPDC proteins in health and disease.

POPDC proteins are a novel class of cAMP-binding proteins, and therefore, the relationship of POPDC proteins to other cAMP effector proteins needs to be defined. In the case of EPAC and PKA, both proteins are often co-localized through the interaction with a common AKAP protein [89,90]. It will therefore be interesting to find out whether POPDC protein are also associated with AKAPs and, thereby, co-localized with EPAC and PKA. Moreover, the biological activity of POPDC proteins in relationship to EPAC and PKA needs to be established. Currently, the role that POPDC proteins play in the nuclear envelope and nucleoplasm is poorly characterized. However, it is interesting that PKA and EPAC1 also have been localized to these compartments [91,92,93].

Thus, future research will need to take into account the complex expression and subcellular localization patterns of POPDC proteins and other cAMP effector proteins. This will provide us with a deeper understanding of the function of POPDC proteins and the physiological effects of cAMP on cellular systems in general.

## Figures and Tables

**Table 1:** Cardiac and skeletal muscle functions of Popdc proteins.

Function	Reference
POPDC genes are predominantly expressed in striated muscle	[1]
*Popdc1^−/−^* mice display a reduction in the number and an increase in the size of caveolae	[33]
*Popdc1^−/−^* mice display an increased vulnerability to ischemia-reperfusion injury	[33]
Popdc1 controls membrane trafficking of TREK-1	[10]
*Popdc1* or *Popdc2* null mutant mice display an age-dependent stress-induced bradycardia phenotype	[10]
Depletion of either *Popdc1* or *Popdc2* in mice results in structural remodelling of SAN tissue	[10]
Depletion of *popdc1* or *popdc2* in zebrafish causes AV-block and muscular dystrophy	[9,12]
Popdc1 in zebrafish controls the formation of the myotendinous junction	[12]
The missense mutation *POPDC1^S191F^* causes cardiac arrhythmia and muscular dystrophy in patients	[12]
*POPDC1* and *POPDC3* are downregulated in human heart failure	[40]
*POPDC1* has been associated with atrial fibrillation	[38]
*POPDC1* is a novel genetic determinant of the QT interval and the QRS time	[39]

**Table 2:** Popdc interaction partners.

Protein	Evidence	Reference
TREK-1	GST-PD, Co-IP, Co-IF, FRET, TEVC	[10]
Caveolin-3	Co-IP, Co-IF	[33]
Dystrophin	Co-IP, Co-IF	[12]
Dysferlin	Co-IP, Co-IF	[12]
VAMP2, VAMP3	Y2H, GST-PD, Co-IF	[49]
GEFT	Y2H, GST-PD, Co-IF	[51]
NDRG4	Y2H, GST-PD, Co-IP, Co-IF	[50]
ZO1	GST-PD, Co-IF, IG-EM	[42]
PR61α	Y2H, Co-IP	[83]
c-MYC	Co-IP, PLA	[83]

Abbreviations: Co-IF, co-localisation by immunofluorescence; Co-IPT, co-immunoprecipitation, FRET, fluorescence resonance energy transfer; GST-PD, glutathione S-transferase pull down; IG-EM, immunogold electron microscopy; PLA, proximity-ligation-assay; TEVC, two-electrode voltage clamp; Y2H, yeast two hybrid.

## Abbreviations

The following abbreviations are used in this manuscript:

AKAP	A-kinase anchoring protein
ANO5	anoctamin 5
AV-block	atrioventricular block
AVN	atrioventricular node
BVES	blood vessel epicardial substance
cAMP	cyclic adenosine monophosphate
CAP	catabolite activator protein
CAV3	caveolin 3
Cdc42	cell division control protein 42 homolog
CFP	cyan fluorescent protein
cGMP	cyclic guanosine monophosphate
cNGC	cyclic nucleotide-gated channel
cNMP	cyclic nucleotide monophosphate
CRIS	cyclic nucleotide receptor involved in sperm function
CRP	cAMP receptor protein
Cx40/43	connexin 40/43
DCM	dilated cardiomyopathy
DGC	dystrophin glycoprotein complex
DMD	Duchenne muscular dystrophy
dpf	days post-fertilization
DYS	dystrophin
DYSF	dysferlin
ECG	electrocardiography
EDMD	Emery-Dreifuss muscular dystrophy
EPAC	exchange protein directly activated by cAMP
FRET	Förster resonance energy transfer
GLUT4	glucose transporter type 4
GST	glutathione-S-transferase
HF	heart failure
IC50	half maximal inhibitor concentration
kDa	kilo Dalton
K2P	two-pore domain potassium channel
LacZ	ß-galactosidase gene
LGMD	limb-girdle muscular dystrophy
MTJ	myotendinous junction
MyoD	myogenic differentiation gene
NCBI	national centre for biotechnology information
NDRG4	N-myc downregulated gene 4
PBC	phosphate binding cassette
PFAM	protein families
PLA	proximity ligation assay
POPDC	Popeye domain containing
PKA	protein kinase A
PP2A	protein phosphatase 2
Rac1	Ras-related C3 botulinum toxin substrate 1
RhoA	Ras homolog family member A
SAN	sinoatrial node
SNARE	soluble N-ethylmaleimide-sensitive- factor attachment receptor
TREK-1	TWIK related K^+^ channel 1
VAMP2/3	vesicle associated membrane protein 2/3
Y2H	yeast-two-hybrid
YFP	yellow fluorescent protein
ZO1	zona occludens protein 1
